# Increasing and More Commonly Refractory *Mycobacterium avium* Pulmonary Disease, Toronto, Ontario, Canada

**DOI:** 10.3201/eid2808.220464

**Published:** 2022-08

**Authors:** Daan Raats, Sarah K. Brode, Mahtab Mehrabi, Theodore K. Marras

**Affiliations:** Toronto Western Hospital, University Health Network, Toronto, Ontario, Canada (D. Raats, S.K. Brode, M. Mehrabi, T.K. Marras);; West Park Health Care Centre, Toronto (S.K. Brode);; University of Toronto, Toronto (S.K. Brode, T.K. Marras)

**Keywords:** nontuberculous mycobacteria, *Mycobacterium avium*, *Mycobacterium xenopi*, treatment, outcomes, tuberculosis and other mycobacteria, bacteria, Canada

## Abstract

In mid-2014, Public Health Ontario Laboratories identified coincident increasing *Mycobacterium avium* isolation and falling *M. xenopi* isolation in the Toronto, Ontario, Canada, area. We performed a retrospective cohort of all patients in a Toronto clinic who began treatment for either *M. avium* or *M. xenopi* pulmonary disease during 2009–2012 (early period) or 2015–2018 (late period), studying their relative proportions and sputum culture conversion. We conducted a subgroup analysis among patients who lived in the Toronto-York region. The proportion of patients with *M. avium* was higher in the late period (138/146 [94.5%] vs. 82/106 [77.4%]; p<0.001). Among *M. avium* patients, conversion was lower in the late period (26.1% vs. 39.0%; p = 0.05). The increase in the proportion of patients with *M. avium* pulmonary disease and the reduction in the frequency of sputum culture conversion is unexplained but could suggest an increase in environmental *M. avium* exposure.

Pulmonary infection with nontuberculous mycobacteria (NTM) is a chronic, often progressive, debilitating disease. Most published data show that the frequency of NTM pulmonary disease (NTM-PD) is increasing worldwide (*1*–*6*), as are its substantial medical costs (*7*,*8*). The cause of this rise has not yet been elucidated. NTM are widespread in the environment but disease is uncommon, suggesting that host susceptibility is critical, although exposure magnitude is also likely key (*9*–*11*). Some observations indicate that the *Mycobacterium avium* complex (MAC) might be a main driver for the increased occurrence of NTM-PD (*2*,*5*,*12*).

In Ontario, Canada, a rising prevalence of NTM-PD has been demonstrated previously, and, in the most recent years that have been studied, that increase was driven largely by an increase in MAC (*2*). More recently, in the spring of 2014, the Public Health Ontario Laboratory (PHOL) observed a sustained increase of >50% in the total number of *M. avium* isolates from pulmonary samples and persons with positive cultures for *M. avium* (*13*). A coincident reduction in *M. xenopi* isolates occurred without change in other NTM species. Curiously, this occurrence was only observed in the city of Toronto and the region immediately north (Regional Municipality of York), located between Lake Ontario and Lake Simcoe, which together encompass an area of 2,392 km^2^ and had ≈4.1 million inhabitants as of 2018. In Ontario, at least 95% of NTM isolates are identified at the PHOL (*14*), permitting population-based study. Laboratory techniques at PHOL did not change at the time of increased isolation. Although the sudden increase in isolation frequency could suggest increased environmental exposure, the reason remains unclear.

Whether and how those changes relate to treatment outcomes of patients with NTM-PD caused by *M. avium* and *M. xenopi* has not been evaluated. In recent years, we observed that patients with *M. avium* pulmonary disease (Mav-PD) more often had microbiologically refractory disease and that we were encountering fewer patients with *M. xenopi* pulmonary disease (Mx-PD). On the basis of those observations, we studied relative proportions, culture conversion, culture reversion, and clinical treatment success of patients with Mav-PD and Mx-PD before and after 2014.

## Methods

We designed this retrospective cohort study to compare patients with Mav-PD and Mx-PD treated before and after 2014. All patients assessed at the Toronto Western Hospital outpatient NTM clinic during July 1, 2003–December 31, 2019, were evaluated for eligibility. The NTM clinic is a tertiary care clinic, usually seeing patients after referral from infectious disease or pulmonary specialists. To be eligible for the study, patients were required to meet the American Thoracic Society/Infectious Diseases Society of America criteria for Mav-PD or Mx-PD (*15*) and to have begun treatment for NTM-PD during January 1, 2009–December 31, 2012, or January 1, 2015–December 31, 2018. These 4-year periods were selected to fall before and after the local surge in *M. avium* isolation and to permit adequate treatment follow-up among all patients in the early period, before the increase in *M. avium* isolation occurred. To be eligible, patients also were required to have been treated for >6 months with >2 antimycobacterial drugs (significant treatment). Patients with non–*M.* *avium* MAC species or those with confirmed macrolide resistance were excluded. Patients with multiple NTM species meeting the microbiologic criterion (*15*) over their clinical course were included, as long as they did not meet the criteria for both Mav-PD and Mx-PD at the start of their treatment. Patients previously treated for NTM-PD with a start date outside of the specified periods were also included, but a 6-month treatment-free interval was required for inclusion. Patients included in the first period were excluded from the second. The study was approved by the University Health Network Research Ethics Board; the need for informed consent was waived.

We collected demographic, clinical, microbiologic, and radiologic data until the first-occurring event of death, loss to follow up, or end of follow-up period. Patients started on treatment during 2009–2012 (early period) were followed until December 31, 2013. Patients started on treatment during 2015–2018 (late period) were followed until December 31, 2019. Follow-up time was measured from treatment initiation to death, loss to follow up, or follow-up end date. Treatment initiation and regimen were decided by the attending physician on the basis of contemporary guidelines.

Baseline clinical characteristics were age, sex, smoking history, comorbidities, body mass index, pulmonary function, corticosteroid use, current or recent chemotherapy, housing, and previous treatment for NTM-PD. Baseline data consisted of recorded observations preceding and closest available to the start of treatment. Significant oral corticosteroid use was use of >7.5 mg prednisone (or equivalent) daily, for >30 days, within the 180 days before the baseline visit (*16*). The predominant radiologic pattern was nodular-bronchiectatic, fibrocavitary, or other, on the basis of computed tomography (CT) results obtained closest to treatment start (*17*). Antibiotics were listed only if prescribed for >3 months for oral or inhaled antibiotics, and only if prescribed for >1 month for intravenous antibiotics, to exclude antibiotics given for concurrent infections or discontinued early because of intolerance.

We assessed treatment outcomes by bacterial species (*M. avium* or *M. xenopi*), comparing patients who started NTM-PD treatment in the early period to those who started in the late period. Culture conversion within 12 months was the primary outcome, defined as >3 consecutive negative mycobacterial cultures collected >4 weeks apart (*18*). We considered the date of the first negative sample to be the date of culture conversion. We assessed the difference in culture conversion rate within and outside of the Toronto-York region in predefined subgroups on the basis of patients’ residential postal codes. Culture reversion was defined as reappearance of the causative species in >2 samples in a patient still receiving treatment who had previously achieved culture conversion. Radiologic evolution was graded as improvement, stability, or progression on the basis of the radiologist’s interpretation and was subsequently used to determine clinical treatment success. We considered patients with symptomatic improvement and at least radiologic stability or patients with radiologic improvement and at least symptomatic stability as demonstrating clinical treatment success. We evaluated that status on the basis of first available data 12 months after treatment initiation. For patients whose treatment was interrupted or incomplete, we used the last available data, but only if they were recorded after >6 months of treatment.

Continuous data were tested for normality through visual inspection and the Shapiro-Wilk test. Most continuous data were not normally distributed, and so we presented all data as medians with interquartile ranges (IQRs) for uniformity. We tested differences between groups by using Fisher exact tests or Mann-Whitney U tests as appropriate and used χ^2^ tests for larger contingency tables. We excluded missing baseline data from respective analyses. We regarded patients with missing data for one of the outcomes as if they had not reached the respective outcome, so that if any bias would be introduced by including these outcomes, it would be bias toward the null. We performed all statistical analyses using GraphPad Prism version 8.4 (https://www.graphpad.com).

## Results

During the study period, 984 patients were assessed in the NTM clinic; MAC or *M. xenopi* were isolated in 880 patients at some point. Significant NTM-PD treatment was started in the specified periods in 301 patients. We excluded 6 patients for not meeting American Thoracic Society/Infectious Diseases Society of America criteria (*15*), 36 patients for having non–*M.* *avium* MAC-PD, 1 patient for potentially having both Mav-PD and Mx-PD, 5 patients because of macrolide resistance, and 1 patient in whom outcomes could not be assessed. A total of 252 patients were eligible.

Among eligible patients, the relative proportion with Mav-PD was higher in the late period (138/146 patients [94.5%]) than in the early period (82/106 patients [77.4%]; p<0.001). The proportion excluded for non–*M.* *avium* MAC-PD remained constant over both periods (data not shown).

We compared the general and NTM disease characteristics of the eligible patients (Tables 1, 2). Among patients with Mav-PD, comorbidities, lung function, and CT pattern were similar between periods. Inhaled corticosteroids were used more often in the early period than the late period (47.6% of patients vs. 26.2%), but this difference was not statistically significant (p = 0.12). Patients in the early period were less likely to have had a positive smear for acid-fast bacilli (64.6% vs. 81.2%; p = 0.01). Patients with Mav-PD were older than patients with Mx-PD (median age 67.6 [IQR 59.2–74.9] vs. 57.4 [IQR 51.8–70.1] years; p = 0.03), had COPD less frequently (68/220 [30.9%] vs. 16/32 [50.0%]; p = 0.04), and less frequently had fibrocavitary disease (51/220 [23.2%] vs. 20/32 [62.5%]; p<0.001).

**Table 1:** Baseline characteristics of patients with *Mycobacterium avium* and *M. xenopi* pulmonary disease, Toronto, Ontario, Canada*

Characteristic	*Mycobacterium avium*				*Mycobacterium xenopi*		
	Early period, n = 82	Late period, n = 138	p value		Early period, n = 24	Late period, n = 8	p value
Median age, y (IQR)	66.3 (59.5–72.7)	68.8 (59.1–76.0)	0.16		57.4 (47.4–72.5)	61.7 (58.2–68.5)	0.51
Sex							
F	54 (65.9)	82 (59.4)	0.39		14 (58.3)	5 (62.5)	Referent
M	28 (34.1)	56 (40.6)			10 (41.7)	3 (37.5)	
Race							
White	57 (69.5)	95 (68.8)	Referent†		20 (83.3)	8 (100)	0.55†
East Asian	22 (26.8)	28 (20.3)			3 (12.5)	0	
South Asian	2 (2.4)	12 (8.7)			1 (4.2)	0	
Black	1 (1.2)	3 (2.2)			0	0	
Smoking history							
Never	46 (56.1)	72 (52.2)	0.56†		8 (33.3)	1 (12.5)	0.39†
Prior	26 (31.7)	53 (38.4)			12 (50.0)	3 (37.5)	
Current	10 (12.2)	13 (9.4)			4 (16.7)	4 (50.0)	
Median BMI, kg/m^2 ^(IQR)	21.1 (18.5–23.2)	21.4 (19.1–24.3)	0.40		21.3 (19.4–24.3)	21.5 (20.7–25.2)	0.35
% Predicted FEV_1_ (IQR)	64.0 (46.5–75.0)	64.0 (46.3–79.8)	0.88		64.0 (45.5–75.5)	80.5 (43.0–95.0)	0.32
% Predicted FVC (IQR)	80.0 (64.8–93.0)	78.0 (65.3–94.0)	0.98		83.0 (68.0–91.0)	101.0 (87.3–109.0)	**0.03**
Comorbidities							
COPD	22 (26.8)	46 (33.3)	0.37		12 (50.0)	4 (50.0)	Referent
Asthma	13 (15.9)	18 (13.0)	0.56		5 (20.8)	3 (37.5)	0.38
Interstitial lung disease	2 (2.4)	6 (4.4)	0.71		0	0	NA
Previous tuberculosis	9 (11.0)	11 (8.0)	0.47		3 (12.5)	0	0.55
Cystic fibrosis or PCD	1 (1.2)	3 (2.2)	Referent		0	0	NA
Previous chest radiotherapy	5 (6.1)	14 (10.1)	0.33		4 (16.7)	0	0.55
Autoimmune disease	14 (17.1)	24 (17.4)	Referent		0	0	NA
GERD	16 (19.5)	38 (27.5)	0.20		6 (25.0)	2 (25.0)	Referent
Aspiration	5 (6.1)	8 (5.8)	Referent		2 (8.3)	0	Referent
Medication use							
Inhaled corticosteroids	39 (47.6)	50 (26.2)	0.12		12 (50.0)	4 (50.0)	Referent
Oral corticosteroids	4 (4.9)	9 (6.5)	0.77		0	1 (12.5)	0.25
Current or recent chemotherapy‡	1 (1.2)	5 (3.6)	0.42		1 (4.2)	0	Referent
Housing§							
Detached single-family	33 (40.2)	52 (37.7)	0.55		12 (50.0)	5 (62.5)	0.53
Attached single-family	16 (19.5)	22 (15.9)			1 (4.2)	1 (12.5)	
Low-rise multi-family	10 (12.2)	12 (8.7)			4 (16.7)	0	
High-rise multi-family¶	23 (28.0)	50 (36.2)			7 (29.2)	2 (25.0)	

*Values are no. (%) except as indicated. Bold indicates significance. BMI, body mass index; COPD, chronic obstructive pulmonary disease; FEV_1_, forced expiratory volume in 1 s; FVC, forced vital capacity; GERD, gastroesophageal reflux disease; IQR, interquartile range; PCD, primary ciliary dyskinesia. †Fisher exact tests comparing White and non-White persons and persons who have ever smoked with persons who have not. ‡Recent chemotherapy was defined as within 2 years of treatment initiation. §Missing data for 2 *M. avium* patients in the late period. ¶Buildings with >5 stories were classified as high-rise.

**Table 2:** NTM disease characteristics of patients with *Mycobacterium avium* and *M. xenopi* pulmonary disease, Toronto, Ontario, Canada*

Characteristic	*M. avium*				*M. xenopi*		
	Early period, n = 82	Late period, n = 138	p value		Early period, n = 24	Late period, n = 8	p value
Previous NTM treatment†							
Same species	10 (12.2)	23 (16.7)	0.47		2 (8.3)	1 (12.5)	Referent
Any species	12 (14.6)	27 (19.6)			5 (20.8)	2 (25.0)	
History of positive AFB smear	53 (64.6)	112 (81.2)	**0.01**		13 (54.2)	5 (62.5)	Referent
CT pattern							
Nodular-bronchiectatic	60 (73.2)	89 (64.5)	0.33		7 (29.2)	1 (12.5)	0.07
Fibrocavitary	17 (20.7)	34 (24.6)			16 (66.7)	4 (50.0)	
Other	5 (6.1)	15 (10.9)			1 (4.2)	3 (37.5)	
CT cavitation, any size	33 (40.2)	57 (41.3)	0.89		18 (75.0)	4 (50.0)	0.22
Median time from initial visit to treatment initiation, mo (IQR)	1.5 (–15.3 to 17.5)	0.0 (–5.0 to 9.0)	0.39		0.5 (–8.5 to 17.3)	0.5 (–1.0 to 3.75)	0.91

*Values are no. (%) except as indicated. Bold indicates significance. AFB, acid-fast bacilli; CT, computed tomography; IQR, interquartile range; NTM, nontuberculous mycobacteria. †All except 1 *M. avium* patient with previous treatment history had a record of a single previous treatment episode; all *M. xenopi* patients with previous treatment history had a record of a single previous treatment episode. P values compare previous treatment for any species

Patients were followed for an average of 40.1 months. Ten patients with Mav-PD left follow up within 12 months of treatment initiation; of those, 8 were treated in the late period. Four patients with Mx-PD left follow up within 12 months of treatment initiation; 3 of those were treated in the early period.

Antibiotic treatment differed somewhat between periods among patients with Mav-PD (Table 3). Rifamycins were used more often in the late period than in the early period (79.0% vs. 62.2%; p = 0.008); fluoroquinolones were used less often in the late period than the early period (13.0% vs. 56.1%; p<0.001). In the late period, 23 patients (16.7%) were started on treatment with 2 drugs, compared with just 2 patients (2.4%) in the early period, but this factor did not result in more frequent treatment changes later. The type of treatment used for Mx-PD was similar in both periods. Treatment duration in the early and late period was comparable for Mav-PD and Mx-PD.

**Table 3:** Antibiotic treatment in early and late period used for patients with *Mycobacterium avium* and *M. xenopi* pulmonary disease, Toronto, Ontario, Canada*

Treatment	*M. avium*				*M. xenopi*		
	Early period, n = 82	Late period, n = 138	p value		Early period, n = 24	Late period, n = 8	p value
Initial treatment							
Macrolide	82 (100)	138 (100)	Referent		23 (95.8)	8 (100)	Referent
Ethambutol	78 (95.1)	126 (91.3)	0.42		21 (87.5)	8 (100)	0.55
Rifamycin	51 (62.2)	109 (79.0)	**0.008**		14 (58.3)	6 (75.0)	0.68
Fluoroquinolone	46 (56.1)	18 (13.0)	**<0.001**		9 (37.5)	3 (37.5)	Referent
IV amikacin	1 (1.2)	2 (1.5)	Referent		1 (4.2)	0	Referent
Other	0	2 (1.5)†	0.53		2 (8.3)‡	0	Referent
Total initial drugs							
2 drugs	2 (2.4)	23 (16.7)	**0.001**		5 (20.8)	1 (12.5)	0.66
3 drugs	67 (81.7)	111 (80.4)			16 (66.7)	5 (62.5)	
>3 drugs	13 (15.9)	4 (2.9)			3 (12.5)	2 (25.0)	
Amikacin added							
IV	20 (24.4)	23 (16.7)	0.62		7 (29.2)	1 (12.5)	0.68
Inhaled only	1 (1.2)	7 (5.1)			2 (8.3)	1 (12.5)	
Treatment adapted	16 (19.5)	36 (26.1)	0.33		11 (45.8)	3 (37.5)	Referent
Treatment intensified	7 (8.5)	15 (10.9)	0.65		9 (37.5)	2 (25.0)	0.68
Median total duration, mo (IQR)	21 (13.3–31.5)	18 (13.0–28.8)	0.38		15.5 (10.8–26.0)	18 (10.8–20.5)	Referent

*Values are no. (%) except as indicated. Bold indicates significance. Drugs were counted toward initial treatment if started within the first 3 months of treatment. Changes in treatment were regarded as treatment adaptations if they took place after the first 3 months of treatment. Treatment adaptations were considered intensification if they resulted in a higher number of drugs used. IV, intravenous. †Clofazimine in 1 patient, inhaled amikacin in 1 patient. ‡Clofazimine in 1 patient, linezolid in 1 patient.

Culture conversion among patients with Mav-PD was less frequent in the late period than the early period (26.1% vs. 39.0%; p = 0.05) (Table 4). Culture reversion to positive after conversion to negative was numerically higher in the late period (30.6% vs. 12.5%; p = 0.09). Culture conversion among patients with Mx-PD was stable between the 2 periods and much higher (21/32 patients [65.6%]) than conversion among patients with Mav-PD (68/220 patients [30.9%]; p<0.001). Although we assumed a failure to culture convert if inadequate samples were submitted, recalculating after excluding patients with incomplete data did not change our comparative outcomes. Clinical treatment success was fairly consistent between periods and species.

**Table 4:** Outcomes of treatment in patients with *Mycobacterium avium and M. xenopi* pulmonary disease, Toronto, Ontario, Canada*

Outcome	*M. avium*				*M. xenopi*		
	Early period, n = 82	Late period, n = 138	p value		Early period, n = 24	Late period, n = 8	p value
Mean duration of follow up after treatment initiation, mo (IQR)	31.0 (19.3–44.0)	31.0 (18.3–39.0)	0.23		28.0 (14.8–53.0)	18.5 (12.8–25.8)	0.32
Culture conversion†	32 (39.0)	36 (26.1)	**0.05**		16 (66.7)	5 (62.5)	Referent
Toronto-York region‡	29/68 (42.6)	26/109 (23.9)	**0.01**		14/19 (73.7)	5/6 (83.3)	Referent
Outside Toronto-York region	3/14 (21.4)	10/29 (34.5)	0.49		2/5 (40.0)	0/2	Referent
Culture reversion	4 (12.5)	11 (30.6)	0.09		1/16 (6.25)	0/5	Referent
Clinical treatment success§	56 (68.3)	88 (63.8)	0.47		15 (62.5)	5 (62.5)	Referent

*Values are no. (%) except as indicated. Bold indicates significance. Culture reversion is presented as no. (% of patients out of those who had culture conversion). Patients with insufficient samples submitted for evaluation of culture conversion were deemed not converted. Patients with missing follow up computed tomography results were considered not clinically successful. †Overall insufficient samples: *M. avium* early period, 19 (23.2%); *M. avium* late period, 23 (16.7%); *M. xenopi* early period, 4 (16.7%); *M. xenopi* late period, 2 (25%). ‡Insufficient samples among Toronto-York region patients: *M. avium* early period, 12 (17.6%); *M. avium* late period, 16 (14.7%); *M. xenopi* early period, 3 (15.8%); *M. xenopi* late period, 0. §Missing computed tomography results: *M. avium* early period, 4 (5.0%); *M. avium* late period, 9 (6.5%); *M. xenopi* early period, 1 (4.2%); *M. xenopi* late period, 1 (12.5%).

## Discussion

After the increase in isolation frequency of *M. avium* in Toronto and the Regional Municipality of York, we observed a rise in the relative proportion of patients treated for Mav-PD at our center. The patients with Mav-PD who were treated after this increase occurred achieved culture conversion less often and had a numerically (but not statistically) higher risk for culture reversion, although their baseline characteristics were comparable and clinical treatment success did not differ.

A sudden population-based increase in the frequency of *M. avium* isolation, as was recently observed in parts of Ontario, has not been reported elsewhere. Meanwhile, we observed a relative increase in treated NTM-PD caused by *M. avium* but not NTM-PD caused by *M.* *intracellulare*. The increase in *M. avium* isolation is broadly consistent with previous observations in Ontario (*14*), parts of the United Kingdom (*12*), Catalonia (*19*) and Hawaii, USA (increased MAC isolation) (*20*), and more specifically in the Netherlands (increased *M. avium* isolation) (*21*). In Queensland, Australia, the magnitude of increase in *M. intracellulare* exceeded that of *M. avium* (*1*). In Madrid, Spain, the isolation rate of *M. avium* was stable, whereas in Belgium the rate of *M. intracellulare* increased and the rate of *M. xenopi* decreased (*22*,*23*). The frequency of NTM-PD and MAC-PD in Denmark was relatively stable during 1997–2008 (*24*), but the prevalence of NTM-PD in Hawaii doubled from 2005 to 2013 (*20*). In addition, in the United States, substantial increases in NTM-PD overall have been identified in Medicare beneficiaries during 1997–2007 (*25*) and in both commercial managed care and Medicare settings during 2008–2015 (*26*). Species-level population-based data for Japan are unavailable, but regional data demonstrated that increases in MAC-PD consisted of increases caused by *M. avium* and *M. intracellulare* in both Nagasaki prefecture (*27*) and Kyoto (*28*).

Although the increase in isolation of *M. avium* observed in Ontario was temporally associated with a higher frequency of Mav-PD in our cohort, this association alone does not demonstrate causality. The lack of changes at the laboratory level suggests a change either in the number of persons with *M. avium* isolation or higher clinician awareness and more investigations (sputum sampling and CT scans). However, no coinciding proportional increase in sputum submission to the PHOL occurred during the increase in *M. avium* isolation (*13*). Although an increase in use of chest CT has been described in Ontario, the increase was nearly linear during 2007–2016 (*29*). Given the lack of obvious detection bias, the abrupt rise in pulmonary *M. avium* isolation is likely reflective of a population-based phenomenon of more persons with *M. avium*–positive sputum, which in turn led to the changes observed in the clinic. However, the evidence remains circumstantial.

For patients with Mav-PD in our cohort, culture conversion in the early period (39%) was lower than the ≈60% that would be expected on the basis of a meta-analysis from 2017 (*30*). Although this difference might be accounted for by the setting and severity of disease, culture conversion in the late period was even lower, occurring in only 26% of patients. In our secondary analysis, we included only patients living in the Toronto and York regions, which resulted in an even more substantial decrease in culture conversion over time. In addition, in the early period, we observed a frequency of culture reversion that was comparable to a large retrospective series (14% microbiologic recurrence on treatment, similar to the definition of reversion used in our study) (*31*), whereas reversion seemed to be more frequent in the late period.

The reduction in culture conversion between periods could be related to treatment regimens. Even though a third drug was more often omitted in the late period, this omission was mostly because of less frequent use of fluoroquinolones, and their effectiveness in treating *M. avium* is debatable (*32*). In addition, rifampin was used more often in the late period, making 3-drug treatment more often in line with current guidelines. Our low overall proportion of culture conversion could be seen as surprising. Accordingly, factors influencing culture conversion, despite not being the primary focus of this study, merited further consideration. In light of the large proportion of patients with cavitation on their CT results, the paucity of injectable amikacin therapy in the initial regimen might suggest inadequate treatment. We generally initiate oral therapy first and allow time for the patient to acclimate and for adjustment of drugs and doses before adding amikacin, which is usually added after ≈12 weeks and thus not classified in our initial 12-week regimen. In addition, we did not employ a minimum-size criterion for cavitation, so a large proportion of patients classified as such likely had small cavities (<2 cm), which might represent foci of bronchiectasis without substantial extrabronchial parenchymal destruction, for which parenteral therapy might not be required. The proportion of patients with Mx-PD who received amikacin was lower still. The reasons for this difference are unclear, although anecdotally, a substantially larger proportion of patients with *M. xenopi* might have declined the recommendation for peripherally inserted central catheter placement and intravenous therapy. On the other hand, acquiring new strains after treatment has begun might explain sputum culture reversion after successful conversion (*33*). Exposure to new strains because of higher levels of exposure might also be a factor in both the overall low conversion rate and the further reduction in the late period.

Because *M. avium* is acquired from the environment, probably more likely from water aerosols than from soil (*9*–*11*,*34*), and higher levels of exposure have been linked with more frequent NTM-PD (*10*,*35*), increased exposure is a plausible mechanism for the changes that were observed in Ontario. Host susceptibility also plays a role in acquiring disease (*6*,*36*) but would be expected to change only gradually over time. Alterations in behavior, such as increased shower use (*10*) or climate changes leading to different surface water microbiome or higher atmospheric water content (*9*), could increase exposure but would not be restricted to such a narrowly defined region. *M. avium* colonizing municipal water and household plumbing might be a substantial source of Mav-PD (*34*). Also, all of the drinking water for Toronto and a large proportion of drinking water for the York region is sourced from Lake Ontario and treated in 1 of 4 water treatment plants, all of which use the same protocols for filtration and disinfection, before the water is pumped northward to consumers in Toronto and much of the York region. Although definitive proof is lacking, changes in the municipal water or its complex distribution system could potentially be causes of increased exposure. This serious public health issue needs additional research, ideally including evaluation of water samples at different sites. Toronto Public Health did not find geographic clustering within Toronto, but whether water testing was performed is unclear (*13*), and Ontario drinking water regulations do not mandate testing for NTM. Other potential confounding factors could not be evaluated in this study.

The consistent clinical treatment success for Mav-PD between periods, despite microbiologic outcomes, is encouraging. It appears that clinical results of treatment in our setting are not exclusively dependent upon culture conversion. For example, patients with more extensive disease at start of treatment might have lower chances of conversion but could still have a good clinical result, presumably associated with a reduction in burden of the organism. We lacked detailed data regarding the burden of organism (i.e., colony counts on solid media) and tried to remediate this shortcoming by looking at sputum smear conversion, but not enough useful data were available. In addition, the possibility of acquisition of new strains of *M. avium* during treatment could not be addressed.

A strength of our study is that comparing outcomes of patients before and after the increase in *M. avium* isolation at the largest NTM referral clinic within the area of increase provides data on a large sample of relevant patients divided over distinct time periods. In addition, by applying broad selection criteria, we were able to include most patients that were treated for NTM-PD caused by *M. avium* or *M. xenopi* at our center, and the number of patients who did not complete follow up was low. 

The first limitation of our study is that several patients in the late period were likely infected before the increase in *M. avium* isolation was observed, because NTM-PD is a chronic disease and treatment initiation might not accurately represent timing of infection. Nevertheless, treatment initiation is undoubtedly related to the infection progressing, which in turn could be influenced by increased exposure, so this approach was most suited to our objectives. Second, patients that were previously treated could have lower conversion rates. Because we excluded patients from the late period who were included in the early period, we expected previous treatment to be more frequent in the early period, and this factor could have reduced the likelihood of detecting a difference in outcomes. However, both Mav-PD groups had comparable levels of previous treatment, so the effect on our results was likely not substantial. Third, we would ideally have studied only patients living in Toronto and the York region, but because we only possessed patients’ addresses at the time of data collection and patients could have moved in or out of the area during treatment, we had to limit this evaluation to a secondary analysis. Last, because sputum samples were collected at the discretion of the treating physician and according to patients’ willingness, the timing and number of samples varied considerably between patients. However, we could not discern any sort of sampling bias that could have influenced the outcomes. We assumed a failure to culture convert if inadequate samples were submitted, but recalculating conversion frequencies after excluding patients with incomplete data did not change our comparative outcomes.

The increased isolation of *M. avium* in Ontario was temporally associated with a higher relative number of patients with Mav-PD, less frequent culture conversion, and a trend toward more frequent culture reversion in Mav-PD patients in our NTM clinic in Toronto. Our findings suggest the presence of a causal relationship between the increased frequency of *M. avium* isolation and clinical events, and by extension, the importance of investigations into the cause and public health consequences of the higher number of *M. avium* isolates.
